# The eating habits of Patients with Type 2 diabetes in Algeria

**DOI:** 10.12669/pjms.322.9266

**Published:** 2016

**Authors:** Aicha Laissaoui, Rachida Allem

**Affiliations:** 1 Aicha Laissaoui, PhD., Laboratory of Natural Bioresources, Department of Biology, Faculty of Science, University of Hassiba Ben Bouali Chlef, Box 151, 02000 Chlef, Algeria; 2 Prof. Rachida Allem, Laboratory of Natural Bioresources, Department of Biology, Faculty of Science, University of Hassiba Ben Bouali Chlef, Box 151, 02000 Chlef, Algeria

**Keywords:** Dietary, Eating habits, Physical activity, Type 2 diabetics

## Abstract

**Objective:**

To evaluate the eating habits and the practice of physical-activity of patients with Tyhpe-2 diabetes. (DT2).

**Methods:**

A total of 1523 patients DT2 with average age 58±9.9 were recruited. A questionnaire about their eating habits, physical activity was conducted. Data were analyzed using SPSS statistical.

**Results:**

Most of the patients were obese (64%), with irregular and weak practice of the physical-activity. The patients based their consumption on food rich with nutrients of high glycaemic index. Their food was mainly characterized by high amounts of fats, the green salads and the desserts (fruits) represent only a secondary amount. Statistically, Overweight + obese patients with diabetes had significantly higher level of consumption of the bread. However, the normal weight patients with diabetes had significantly higher level of the consumption of fruit and vegetables (p=0.006 and p=0 respectively). On the other hand, there was no significant difference in level of the consumption of the greasy substances, milk and dairy products, meat-fish-egg two groups (p=0.53, p=0.06 and P > 0.05).

**Conclusion:**

This study showed the need for an improvement in the nutritional care of DT2 patients in the area of Ain Defla (Algeria), also, the practice of the physical-activity, in order to plan an adequate therapeutic care.

## INTRODUCTION

The prevalence of the type 2 diabetes is higher in the world, especially among patients coming from the developed countries, like the West, the Middle East and sub-Saharan Africa.^1^ The hygiene-dietetic rules represent the base of the care for the type 2 diabetes; they aim not only the improvement of the glycaemia, but also the risk factors frequently associated with the diabetes.^2^

The physical-activity forms integral part of the therapeutic assumption of responsibility of diabetes of the type 2.^3^ The interventions that target the physical activity level of an individual and his food are essential elements of the management of type 2 diabetes.^4^ The people with diabetes must take care to have a food varied and balanced.^5^ The nutritional care of a type 2 diabetic patient consists on the knowledge of the relation between nutrients, food, weight and the insulin-resistance.^6^

The objective of this study was to evaluate the dietary habits and the practice of the physical-activity at a certain number of diabetic inhabitants in the area of Ain Defla in order to propose adequate preventive plans.

## METHODS

A cross-sectional study was conducted at Ain Deflain the west province of Algeria during the period from March to December 2011. A total number of 1523 patients with diabetes of the Type-2 (988 women and 535 men) participated in the study. This study was conducted in accordance with the declaration of Helsinki with approval from the Ethics Committee of our Hospital. Written informed consent was obtained from all participants. Each patient had undergone an interrogation including the clinical characteristics (age, sex, duration of diabetes evolution, Body Mass Index (BMI), waist measurement, blood pressure) and a food consumption survey helped to reconstitute, the eating habits. The questionnaire comprised also items relied on: physical activity. Our diabetics were divided according to BMI: Group 1 (G1): normal weight diabetics, group 2 (G2): overweight + obese diabetics’.

### Data Analysis

Statistical analysis was performed using SPSS version 17. Independent samples t-test was used to compare means of different variables. Data were presented as mean ± standard deviation (SD). The results were considered statistically significant when the two tailed *p* value was < 0.05.

## RESULTS

Out of 1523 patients with type 2 diabetes included in this study, 988 were female and 535 were men. The average age of the patients was (58.7 ± 9.9) years respectively. The intermediate duration of the diabetes was (10.5 ± 5.1) years. Most of the patients were obese (64%), the women had a higher body mass index than men. 60% of the patients were hypertensive.

### Food habits

The bread, Potato as well as the greasy substances consumption is very high in the diabetics with overweight+obese (191.12±40.62g/day, 5 times/week, 28.65±7.25g/day) respectively compared to normal weight diabetics (167.77±24.87 g/day, 4 times week, 20.42 ± 5.57 g/d) followed by the consumption of fast sugars, on the other hand a low consumption of milk and dairy product in the two groups. The diabetics consumed less meats and fish in the two groups (Table-I).

**Table-I:** Quantitative estimation of various foods for the two groups of patients.

Types of food	Quantity of food consumed	

	Group 1 Normal Weight Diabetics	Group 2 Overweight + Obese diabetics
Bread (g/day)	167.77±24.87	191.12± 40.62
Cereals	3 times/week	2 times/week
Leguminous plants	2 times/week	1times/week
Potato	4 times/week	5times/week
Fruit and vegetables	3-5 fruit and vegetables/day	2-4 fruit and vegetables/day
Milk and dairy products (g/day)	194.52 ± 23.05	200.55 ± 12.34
Meats	3 times/week	once a week
Fish	once a month	once a month
Eggs	2 eggs/week	3 eggs/week
Greasy substance (margarine, olive oil, butter…) (g/day)	20.42 ± 5.57	28.65 ± 7.25
Viennoiseries, pastry makings (growing in particular)	2 times/week	3 times/week
Sweetened products (Chocolates, Honey, Confectioneries, sugar of table…) (g/day)	22.37 ± 3.84	53.24 ± 12.14
Drinks sweetened (Soda, juices industrial…) (ml/day)	150 ± 24.56	230.21 ± 33.77
Water (l/day)	1.3 ± 0.3	1.15 ± 0.25

### Carbohydrates consumption at the two studied groups

The complex carbohydrates were consumed daily by patients but with different proportions; 77% for normal weight diabetics and 71% for overweight+ obese diabetics. The simple carbohydrates and complex carbohydrates were consumed daily by the two groups but with different proportions is 23% and 77% at the Group-1 and 29% and 71% at Group 2 respectively, the difference was significant between the two groups.

### The bread consumption by both groups

Compared with the normal weight diabetics, the bread consumption was high and there was a significant difference (P=0.006) (Fig-I).

**Fig.1:**
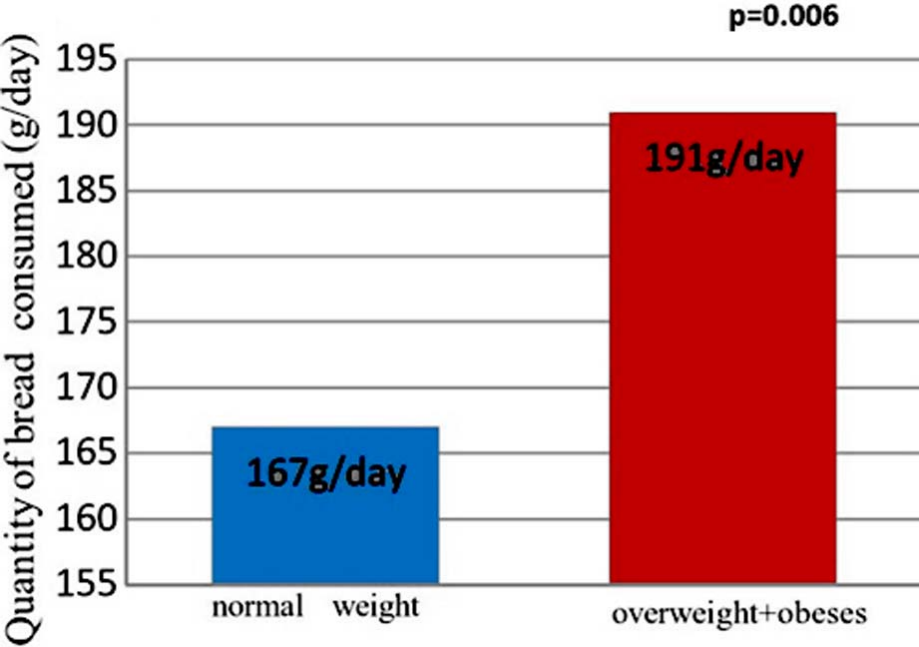
Quantity of bread consumed by both groups of patients with Type-2 diabetes.

### The consumption of the greasy substances by both groups

The normal weight diabetics consumed more unsaturated greases and less saturated greases than group 2. However, the difference was not significant between the two groups (p=0.53) (Fig.3).

**Fig.2:**
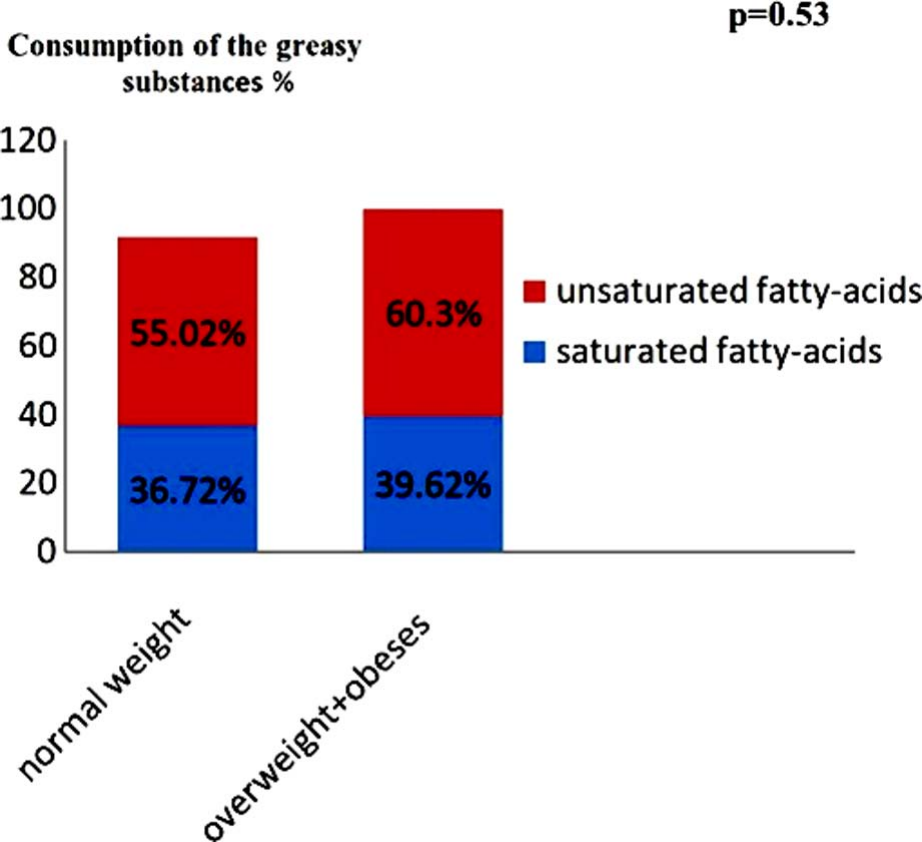
Consumption of the greasy substances at the two groups in patients with Type-2 diabetes.

**Fig.3:**
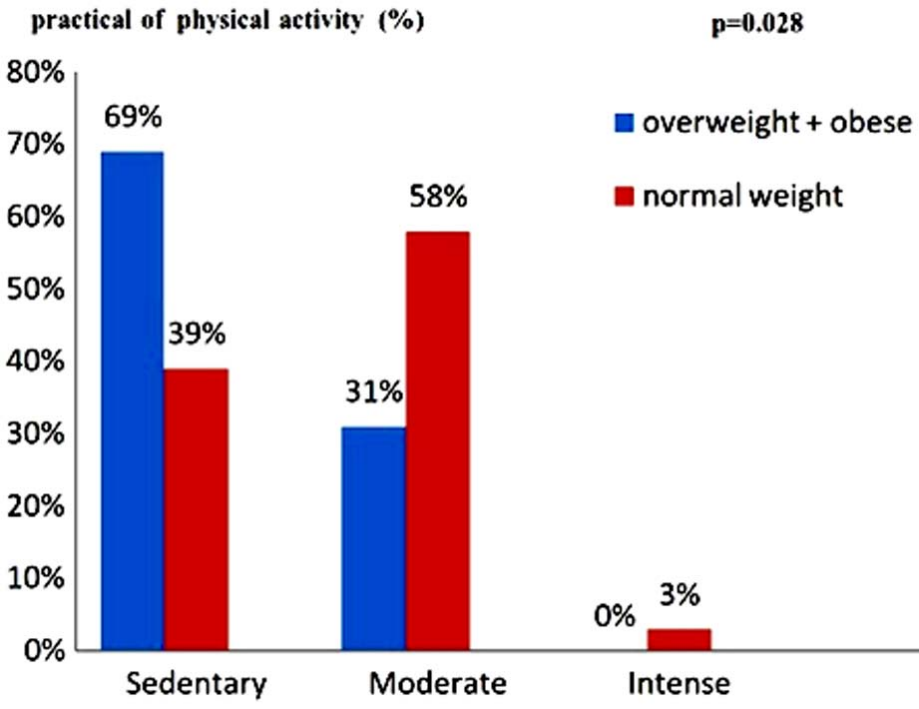
Practical of physical activity in the two groups in patients with Type-2 diabetes..

### The consumption of fruit and vegetables by both groups

Compared with the overweight+ obese diabetics, the consumption of fruit and vegetables was high 492.74g/day in the normal weight patients with diabetes and there was a significant difference in the normal weight patients with diabetes as compared to overweight patients with diabetes 421.84g/day in overweight and obeste patinets.

### The consumption of proteins by the both groups

### Average consumption of milk and dairy products

Compared with the normal weight diabetics, the average of consumption of the dairy products was high in overweight+ obese patients with diabetes 200.55g/day as compared to 194.52g/day in patients with normal weight. The difference was not significant.

### Average meat-fish-egg consumption

The weekly consumption of the MFE (meat-fish-egg) is relatively low at normal weight diabetics compared with overweight+ obese diabetics, with no significant difference (P > 0.05) (Table-II).

**Table-II:** Comparison of the meat-fish-egg consumption in the two groups of diabetics patients type 2.

	The weekly consumption of the meat-fish-egg (g/week)		

	Egg	Meat	Fish
Normal weight diabetics	100±32	320±83	20±3
Overweight + obese diabetics	150±45	360±99	30±10
P value	0.060	0.080	0.082

### Characteristics of the Algerian meals

For most of the patients, the meal of the day comprises a breakfast, a lunch, a snack and a dinner.

- At 8:00 am: a breakfast containing croissants, Algerian pie (equivalent of crepes) or of bread (100 G) with butter and Coffee with milk;
- At 12:00 pm; the lunch is represented primarily by the Algerian traditional, rich in potatoes (it occupies the essential dish of their meal in particular the fries), oil (3 to 4 big Spoons by dishes), the couscous (9 to 12 big Spoons). The bread represented the essential base of the meal (200g). The green salads and the desserts (fruits) represented only a secondary contribution.
- The Snack, around 4:00 p.m., also rich in bread (100g) and jam (1 spoon with soup), and the coffee with milk.
- Dinner at 8 p.m., resembles to lunch, it containing pastes, potato or vegetable soup, but is always accompanied with bread (200g).

### The quantitative and qualitative energy distribution of the diet

The average calorie intake was 2668 ± 320 kcal / day.

### Carbohydrate intake

The average carbohydrate intake represented 55±10% of total calories. Complex carbohydrates accounted for 80% of carbohydrate intake, diet rich in bread (150 g / day / person on average) in potato (120g / day / person; including French fries), couscous with raisins. Mbeses with dates (Traditional cakes based semolina, date, butter and honey).

### Protein intake

The protein intake accounted for 13±3% of total calories. It was characterized by an average consumption of meats, white meats (chicken), eggs, butter, green vegetables and low intakes of fish, red meat and dairy products. Qualitatively, Intake animal / vegetable protein proteins represent = 1.4.

### Fat intake

The fat intake represented 30±7% of total calories. Unsaturated fatty acids provide most of the lipid ration polyunsaturated fatty acids: 49±20%, provided mainly by vegetable oils used in making chips, seasoning, the traditional dishes (Algerians msemen, Algerians mebeses). The dishes (2-4 spoons vegetable oils / dish); monounsaturated fatty acids: March 1±14% provided by olives and olive dish. Saturated fatty acids accounted for 37± 66% of the lipid ration, made mostly by beef, couscous with butter and cold cuts.

### Other nutrients

Dietary fibers provide a contribution of (26±3g/d). However, inadequate calcium intake was found. Intakes of vitamin C and E are insufficient compared to that vitamin of group D and B.

### Physical activity level (PAL) and type 2 diabetes

At normal weight patients with diabetes with sedentary life style accounted for 39%, those with pal moderate 58% and those with intense PAL was 3%. In overweight + obese diabetics, the sedentary accounted for 69%, those with moderate PAL represented 31% and those with intense PAL are 0%. The difference between the two groups was significant (p =0.028) (Fig-III).

## DISCUSSION

The principal food anomalies found in our study are an over-sugar food, rich in food with high glycemic index (bread consumption was too high “191.12± 40.62g/day in overweight + obese diabetics and 167.77±24,87 g / day in normal weight diabetics”, couscous and the potato) disturbing the glycemic balance.

In 2002, Walter Willett et al indicate that the glycemic index and the glycemic load of the overall diet have been associated with a greater risk of type 2 diabetes. Conversely, a higher intake of cereal fiber has been consistently associated with lower diabetes risk. In diabetic patients, replacing high-glycemic-index carbohydrates with a low-glycemic-index forms will improve glycemic control and these dietary changes, which can be made by replacing products made with white flour and potatoes with whole-grain, minimally refined cereal products, have also been associated with a lower risk of cardiovascular disease and can be an appropriate component of recommendations for an overall healthy diet.^7^ an other hand, our diabetics are characterized by a high consumption of drinks sweetened (150±24.56 versus 230.21±33.77 ml/day). The consumption of soft drinks, fruit juice, sweetened-milk beverages and energy from total sweet beverages was associated with higher type 2 diabetes risk independently of adiposity.^8^

The meals of our patients had lot of fats (primarily mono-unsaturated fatty-acids: because of excessive consumption of French fries, couscous with butter. This contributes with sedentary life style leading to maintain obesity. The Saturated fatty acids accounted for 37±66% of the lipid rations, (36.72% normal weight diabetics, 44.97% of overweight+ obese diabetics) on our diabetics, these fatty-acids are correlated positively with the hyper LDL cholesterolemia and the cardiovascular complications. Current recommendations for the general population to consume fats is in the range of 20% to 35% of energy intake apply equally to people with diabetes. As the risk of coronary artery disease in people with diabetes is 2 to 3 times that of those without diabetes, saturated fats should be restricted to <7% of total daily energy intake.^9^ On the other hand, the unsaturated mono fatty-acids are negatively correlated with the hyper LDL-cholesterolemia. Qualitatively, food intake showed several anomalies which subject our diabetics to the risk of cardiovascular complications citing high: report animal protein / vegetable = 1.4.a saturated fatty acid / polyunsaturated> 1; Inadequate intake of antioxidant vitamins.

The fruit and vegetables consumption corresponds to 492.74± 33.36 g/day for normal weight diabetics and 421.84±32.23 g/day for overweight+obese diabetics. As recommended by the World Health Organization, it is very healthy to take at least 5 fruit and vegetables per day, for their contribution in vitamins, minerals and food fibres’.^10^ A consumption of more than 3 or 5 fruit and vegetables per day is associated with a reduction of 1.010 to 0,962 risk of diabetes, in comparison with a consumption lower than these thresholds respectively.^11^ The increasing consumption of green leafy vegetables of about one serving per day was associated with a statistically significant 14% reduction in the risk of type 2 diabetes.^12^

The practice of a physical-activity was weak within our patients (Fig.6). This weak practice could be explained by sociocultural factors: Most (64.87%) of the subjects were relatively elderly women and without any work. Therefore, we can undoubtedly add the ignorance of the beneficial effect of the physical-activity in the care for the diabetes and obesity,^13^,^14^ as well as time average sitting in front of television (200±55 min/day) and on Internet (45± 9 min/day).

A unique finding in the present work was the significant positive correlation between the bread; potatoes and BMI. The potatoes and particular the bread are the both foods consumed by Algerians as essential food for each meal. It is well known that consuming the white bread lower (white bread was consumed by our diabetic) satiety and increases energy intake at the next meal. That consumption of whole-grain bread is more beneficial than refined bread, especially in relation to abdominal fat distribution.^15^

## CONCLUSION

This study identified food habits of patient’s type 2 diabetics in Ain Defla (Algeria). It highlights the need for the change in mode living with diabetes, and especially the need to develop means and tools for nutrition education, as the nutrition is the cornerstone of the appropriate management of diabetes. Also, the practice of the physical-activity, in order to plan an adequate therapeutic care.
